# Higher Education and Nurses' Needs

**Published:** 1918-11-30

**Authors:** 


### THE MATRONS' AND SISTERS' DEPARTMENT.

### HIGHER EDUCATION AND NURSES' NEEDS.

The College of Nursing has not so far officially given a definite lead to the movement within the profession tending towards shorter hours and better pay. But we have often pointed out that both these aims have been kept strongly in view by the nurses composing the Council of the College, who have experimented within their own domains and demonstrated the feasibility of reforms which, while they were on paper only, may well have seemed outside practical consideration. In this indirect way the College, through the governing body charged with its interest, has begun to show the way. Since many nurses impatiently look for some manifestation of policy which will reveal official sympathy with the reformers, we propose to show that the educational reforms the College aims at initiating do in effect entail both a reduction of working hours and an all-round rise of salaries.

Nothing has been more discreditable to the training-schools in the past than the place accorded in the time-table to the theoretical instruction of the probationer in nursing lore. Examinations have too often been carefully designed to let through their easy meshes any and all who should make an effort to answer the questions. Conscientious and intelligent women, desirous of mastering their subjects, received scanty encouragement, and literally no time outside their normal routine in which to study. This state of things is going on now in countless establishments which do not even boast a class-room, and where the only possible way of studying is to take the time out of that assigned to exercise and rest.

A complete transformation takes place in an institution offering facilities for serious study to its probationers. Off-duty time is at once largely increased. The probationer becomes a student, towards whom the institution has the same measure of responsibility as a College towards its undergraduates. No sooner is the theoretical branch of nursing restored to its right place than the ceaseless ritual of the ward falls likewise into its right place. The mind of the probationer rises to the surface, and once that takes life and begins to grow it becomes necessary to feed it. The whole current of the training-school quickens. Stagnation disappears. And the time-table undergoes a revision which is imperatively demanded by the facts of the case. Higher education for probationers inevitably means increase of staff, shorter working hours, more facilities for recreation. The experiment has, we know, been tried of giving longer hours off duty without any stimulus such as results from a keen educational system. The result is disappointing, and almost seems to justify those who contend that nurses do not need so much time to themselves. For, if care is not taken, the centre of interest may be shifted away from the hospital to some other focus, whether it be family preoccupations or amusement and excitement pure and simple. Hence if longer hours off duty are to bring benefit to probationers in any real sense, if they are to help them to a better understanding of their profession in its widest aspects, improve their outlook and render them more efficient all round, there must be a compelling power in their training-school which draws their hearts and minds into tune with their profession.

In taking its stand on higher education for the probationer the College of Nursing is building more wisely than its members can guess. It is opening the gate to a new profession, in which all the old ideals will be safeguarded, while the interests of the patients become the paramount interest of the student. For the life of the student is not by itself entirely satisfying to one women out of a hundred. The centre of a woman's being is her heart, and while she is isolated from human interests, while she is denied the opportunity of offering herself in service, she is living a maimed existence. In the double life of study and service to the sick she finds the bracing atmosphere in which all her faculties may be evenly developed.

